# Stress induces major depressive disorder by a neutral sphingomyelinase 2-mediated accumulation of ceramide-enriched exosomes in the blood plasma

**DOI:** 10.1007/s00109-022-02250-y

**Published:** 2022-08-31

**Authors:** Fabian Schumacher, Alexander Carpinteiro, Michael J. Edwards, Gregory C. Wilson, Simone Keitsch, Matthias Soddemann, Barbara Wilker, Burkhard Kleuser, Katrin Anne Becker, Christian P. Müller, Johannes Kornhuber, Erich Gulbins

**Affiliations:** 1 Department of Molecular Biology, University Hospital Essen, University of Duisburg-Essen, Hufelandstrasse 55, 45122 Essen, Germany; 2 Institute of Pharmacy, Freie Universität Berlin, Königin-Luise-Str. 2+4, 14195 Berlin, Germany; 3 Department of Surgery, University of Cincinnati College of Medicine, Cincinnati, OH USA; 4 Department of Psychiatry and Psychotherapy, University Clinic, Friedrich-Alexander-University of Erlangen-Nuremberg, Schwabachanlage 6, 91054 Erlangen, Germany; 5 Centre for Drug Research, Universiti Sains Malaysia, 11800 Minden, Penang, Malaysia

**Keywords:** Major depression, Neutral sphingomyelinase 2, Ceramide, Exosomes, Behavior, Neurogenesis

## Abstract

**Abstract:**

Major depressive disorder (MDD) is a very common, severe disease with a lifetime prevalence of ~ 10%. The pathogenesis of MDD is unknown and, unfortunately, therapy is often insufficient. We have previously reported that ceramide levels are increased in the blood plasma of patients with MDD and in mice with experimental MDD. Here, we demonstrate that ceramide-enriched exosomes in the blood plasma are increased in mice with stress-induced MDD. Genetic studies reveal that neutral sphingomyelinase 2 is required for the formation of ceramide-enriched exosomes in the blood plasma. Accordingly, induced deficiency of neutral sphingomyelinase 2 prevented mice from the development of stress-induced MDD. Intravenous injection of microparticles from mice with MDD or injection of ceramide-loaded exosomes induced MDD-like behavior in untreated mice, which was abrogated by ex vivo pre-incubation of purified exosomes with anti-ceramide antibodies or ceramidase. Mechanistically, injection of exosomes from mice with MDD or injection of ex vivo ceramide-loaded microparticles inhibited phospholipase D (PLD) in endothelial cells in vitro and in the hippocampus in vivo and thereby decreased phosphatidic acid in the hippocampus, which has been previously shown to mediate MDD by plasma ceramide. In summary, our data indicate that ceramide-enriched exosomes are released by neutral sphingomyelinase 2 into the blood plasma upon stress and mediate stress-induced MDD.

**Key messages:**

- Stress induces ceramide-enriched exosomes in the blood plasma.
- Ceramide-enriched exosomes mediate major depressive disorder (MDD).
- Deficiency of neutral sphingomyelinase 2 protects from stress-induced MDD.
- Neutralization or digestion of ceramide in exosomes prevents stress-induced MDD.
- Ceramide-enriched exosomes inhibit endothelial phospholipase D in the hippocampus.

## Introduction

Major depressive disorder (MDD) is a severe and chronic disease with a lifetime prevalence of more than 10% [1]. The main symptom is a depressed mood, but the disease is also characterized by a loss of interest, anhedonia, fear, feelings of worthlessness, weight loss, insomnia, and concentration deficits [1]. Because approximately 10% of MDD patients attempt suicide, MDD is also often a life-threatening or even fatal illness [1]. In addition, MDD and depressive traits were also associated with many somatic symptoms, such as an increased incidence of cardiovascular disease and osteoporosis, adrenocortical activation, increased oxidative stress, and increased plasma concentrations of proinflammatory cytokines and phospholipase A_2_, as well as dyslipoproteinemia [2–5]. In particular, the study by Kalinichenko et al. demonstrated in a large population of more than 40,000 people this association of somatic symptoms with MDD [5]. These somatic symptoms cannot be explained by exclusive alterations of the central nervous system (CNS) and suggest that MDD can be seen as a systemic disease.

Classic antidepressants, sleep deprivation, electroconvulsive therapy, or ketamine are mainly used to treat MDD [1]. However, classical antidepressants require 2–4 weeks of treatment before showing a therapeutical effect, which is a serious clinical problem. In addition, they are only effective in 60–70% of the patients, often requiring repeated treatments with other antidepressants.

The pathogenesis of MDD is still largely unknown and several hypotheses have been suggested: Since classical antidepressants were previously shown to target the uptake of monoamines, it was hypothesized that MDD is caused by a lack of monoamine transmitters in the synapses, which is corrected by antidepressants [6]. However, although monoamine transmitters seem to be very important in MDD, this hypothesis was revised because some antidepressants, e.g., tianeptine even enhance serotonin reuptake [7]. Furthermore, the delayed action of these drugs with a delay of 2–4 weeks does not fit their immediate inhibition of monoamine transmitters. In addition, Ketamine, which has been recently introduced for treatment of MDD, targets N-methyl-D-aspartate (NMDA) receptors [8, 9], but not the uptake of monoamine neurotransmitters. In addition, since chronic stress and major depression result in inhibition of neurogenesis and hippocampal atrophy, which are reversed by 2 to 3 weeks of treatment with antidepressants [10–13], neurogenesis was suggested as cause of MDD [10–13]. However, other studies showed that inhibition or ablation of neurogenesis, e.g., by selective irradiation of the hippocampus, does not result in MDD in mice [11]. Furthermore, electroconvulsive therapy, an alternative treatment for MDD, and ketamine exert a rapid therapeutic effect in MDD [14], a finding that is also inconsistent with an extended length of neurogenesis and maturation as pathogenetic cause of MDD and requirement for therapy with antidepressants.

Thus, several other hypotheses were tested to explain the pathogenesis of MDD. It was suggested that alterations in growth factors and their receptors, an over-activity of the glucocorticoid system, an increase of inflammatory factors such as interleukin-6 in the brain and/or distinct dysfunctions of endothelial cells in the brain, mediate the pathogenesis of MDD [e.g., 1, 15–20].

However, none of these hypotheses provided a comprehensive model for the pathogenesis of MDD. It seems very likely that MDD is not induced by a single mechanism, but it is rather a combination of factors that triggers MDD. It is also likely that pro- and anti-depressive factors and mechanisms balance each other and that often several factors act together to cause MDD in humans, while under circumstances in which one factor is massively changed, the alteration of this factor or a few factors might be sufficient to mediate MDD.

We have previously shown that antidepressants target the acid sphingomyelinase [21–23]. The acid sphingomyelinase hydrolyses sphingomyelin to ceramide in lysosomes and also on the extracellular leaflet of the plasma membrane [24–26]. Most classical antidepressants are weak bases that are protonated and thereby accumulate in acidic compartments [21, 22, 27–30]. Therapeutic concentrations of the antidepressants such as amitriptyline and fluoxetine reduce acid sphingomyelinase activity and increase sphingomyelin concentrations in the hippocampus and thereby increase neuronal proliferation, maturation, and survival and normalize behavior in models of stress-induced depression [21–23]. The increase of sphingomyelin in lysosomes upon treatment with antidepressants requires 14–21 days, consistent with the time course of the antidepressive action of antidepressants. Increased lysosomal sphingomyelin concentrations induced autophagy in hippocampal neurons, a process, which is greatly reduced in neurons from stressed/depressed mice [23]. However, although mice genetically deficient for the acid sphingomyelinase failed to respond to classical antidepressants, proving the role of this enzyme for the action of these drugs, these mice developed symptoms of MDD upon stress, suggesting that the acid sphingomyelinase does not contribute to the pathogenesis of MDD.

However, although these studies clearly show a role of sphingolipids and in particular the acid sphingomyelinase for treatment of MDD, they did not address the function of sphingolipids in the pathogenesis of MDD. It is important to note that drugs that are used to treat MDD may not interfere directly with molecular mechanisms that cause MDD but may change the balance between several molecular systems in the central nervous system thereby indirectly resulting in a correction of depressed behavior.

Several recent studies indicated an increase of ceramide in the blood plasma of human individuals and mice with MDD [31–33]. We demonstrated that increased blood plasma ceramides inhibit phospholipase D (PLD) in endothelial cells of the hippocampus, resulting in a decrease of phosphatidic acid in the hippocampus [33]. Intravenous injection of anti-ceramide antibodies to neutralize ceramide, ceramidase to consume ceramide, PLD to reconstitute endogenous phosphatidic acid, or phosphatidic acid itself rescued depressed mice and rapidly, i.e., within 24 h, normalized behavior and neuronal proliferation indicating the significance of this novel pathway for MDD. It is important to note that these findings suggest that a peripheral, blood plasma factor induces a central nervous disease, i.e., MDD. Here, we aimed to define the source of peripheral ceramide in the blood plasma upon application of stress. In particular, we tested the role of neutral sphingomyelinase 2 in MDD. Neutral sphingomyelinase 2 has been shown to be critically involved in the cellular release of ceramide-containing exosomes and it is therefore tempting to speculate that this enzyme mediates the increase of ceramide in the blood plasma upon application of stress.

## Results

### Ceramide is increased in the blood plasma in mouse models of MDD

**Fig. 1:**
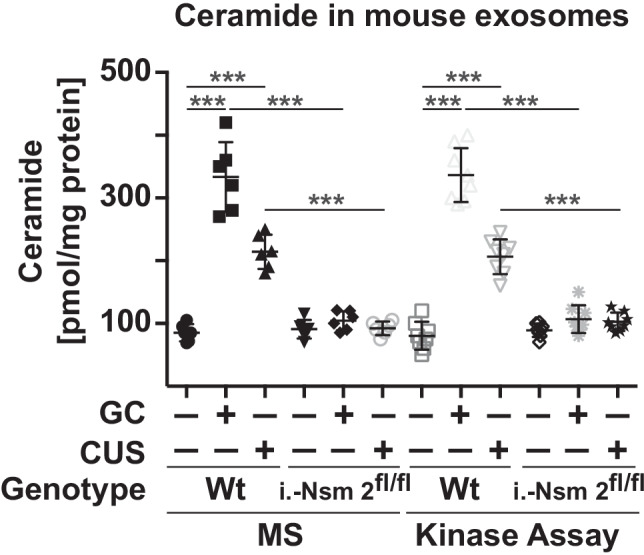
Ceramide levels in exosomes are higher in stressed mice than in control subjects. Wild-type mice or mice induced for deficiency of neutral sphingomyelinase 2 (i.-Nsm 2^fl/fl^) were stressed with either glucocorticosterone (GC) or chronic unpredictable environmental stress (CUS) for each 7 days or were left untreated as controls (-). Venous blood samples were collected at day 7, and exosomes were purified from the blood plasma by ultracentrifugation. Ceramide concentrations (expressed as sum of C16 to C24 sub-species) were determined by ceramide kinase assays and mass spectrometry (MS). Ceramide levels in the exosomes of wild-type mice after various forms of stress were higher than those in the plasma from control subjects. Induced deficiency of neutral sphingomyelinase 2 prevented the increase of ceramide levels in exosomes after stress. Deletion of neutral sphingomyelinase 2 was achieved by injection of tamoxifen 4 weeks prior to the studies. Shown are the mean ± SD, *n* = 6–8 for all mouse samples and *n* = 16–20 for all human samples. ***P < 0.001, ANOVA and post hoc Tukey test

Major depressive disorder is often caused by stress. We have previously shown that stress induces an accumulation of ceramide in the blood plasma, which finally mediates MDD [33]. Here, we tested the hypothesis that exogenous stress induces the release of ceramide-containing microparticles/exosomes into the blood and that neutral sphingomyelinase 2 is mediating this event. We measured the concentration of ceramide-containing microparticles/exosomes in the blood plasma of mice that were exposed to glucocorticosterone-induced or chronic unpredictable environmental stress or left unstressed. We found that glucocorticosterone-mediated or chronic unpredictable environmental stress induced a marked increase in ceramide concentrations in exosomes in the blood plasma of mice (Fig. 1).

Neutral sphingomyelinases have previously been shown to be crucial for the release of exosomes and might mediate the release of ceramide-enriched exosomes [34]. To test the role of the neutral sphingomyelinases in stress responses, we used mice with an inducible deletion of neutral sphingomyelinase 2 (deletion of the neutral sphingomyelinase 2 was induced by Cre-recombination in adult mice). The results demonstrated that ceramide concentrations in the exosomal fraction of mice deficient in neutral sphingomyelinase 2 did not increase (Fig. 1) indicating a central role of neutral sphingomyelinase 2 for the release of ceramide into the blood plasma upon stress.

We also measured the numbers of exosomes in the plasma using an anti-CD63-based ELISA. The results show that glucocorticosterone and CUS treatment did not significantly change the number of exosomes in the blood plasma, i.e., 0.9 × 10^11^ ± 0.22 × 10^11^ exosomes/mL blood plasma in untreated mice, 1.4 × 10^11^ ± 0.3 × 10^11^ exosomes/mL blood plasma after glucocorticosterone stress, and 1.2 × 10^11^ ± 0.25 × 10^11^ exosomes/mL blood plasma after CUS.

### Ceramide-enriched exosomes in the blood plasma are sufficient to induce symptoms of MDD in mouse models

**Fig. 2:**
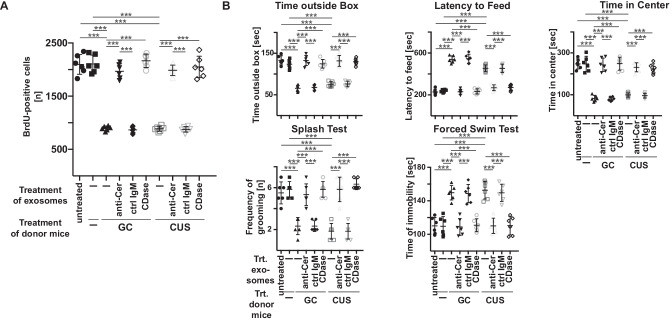
Injection of exosomes from stressed mice induces symptoms of major depressive disorder, which is prevented by ex vivo neutralization or consumption of ceramide. **A** and **B** Exosomes were purified from the blood plasma of untreated or mice that were stressed for 7 days (donor mice). Exosomes were treated ex vivo with ceramide IgM antibodies clone S58-9 (anti-Cer), control immunoglobulin M (ctrl IgM), or recombinant ceramidase (CDase) or were left untreated (-), pelleted, resuspended in H/S, and injected intravenously (i.v.) into healthy wild-type mice. Controls were left completely untreated (-) or were injected with exosomes from untreated mice. Neurogenesis in the hippocampus **A** and behavioral changes **B** were determined 24 h later as readouts for MDD. Shown are the mean ± SD from each 6 animals analyzed per group. ***P < 0.001, ANOVA and post hoc Tukey test

To determine the significance of ceramide-enriched exosomes in the blood plasma for the development of MDD, we purified exosomes from wild-type mice that were exposed to glucocorticosterone or chronic unpredictable environmental stress for each 7 days or left untreated. We incubated the purified exosomes with anti-ceramide antibodies, ceramidase, or control IgM ex vivo or left the samples untreated. After an incubation time of 60 min, we separated the exosomes from any unbound antibody or ceramidase or remaining glucocorticosterone by ultracentrifugation and injected them intravenously into untreated wild-type mice. As indicators of MDD, we determined behavior parameters and neuronal proliferation. The results show that exosomes purified from stressed mice reduced neuronal proliferation (Fig. 2A). Treatment of exosomes from stressed mice with anti-ceramide antibodies or ceramidase before intravenous injection abolished this effect (Fig. 2A). Furthermore, exosomes purified form stressed mice induced depressed behavior within 24 h (Fig. 2B). Incubation of exosomes with anti-ceramide antibodies or ceramidase before intravenous injection prevented development of MDD as indicated by behavioral tests (Fig. 2B). Control IgM did not affect the induction of MDD by exosomes from stressed animals (Fig. 2A and B). Exosomes from untreated animals exerted no effect on behavior and neurogenesis (Fig. 2A and B).

### Injection of ex vivo loaded ceramide-enriched exosomes induces MDD

**Fig. 3:**
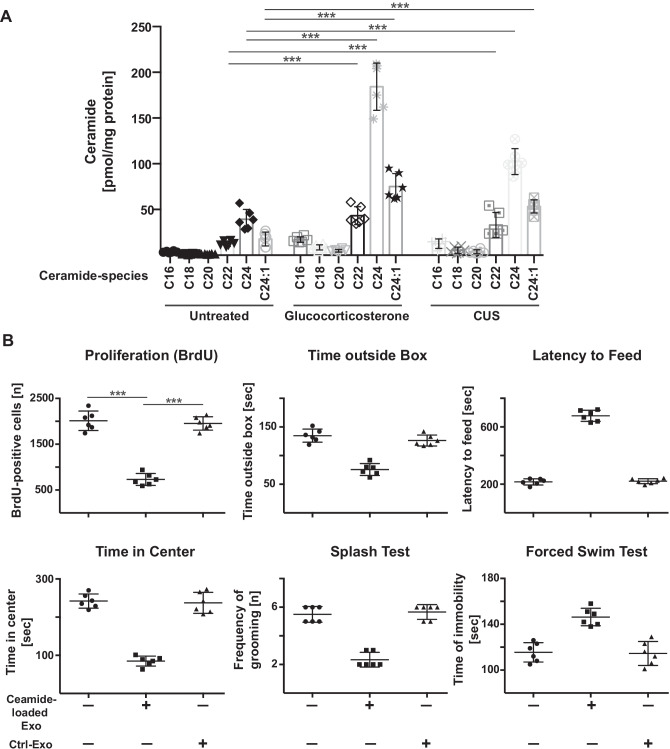
Injection of ceramide-loaded exosomes induces symptoms of major depressive disorder. **A** Exosomes were isolated from the blood of untreated glucocorticosterone- or chronic unpredictable stress-treated wild-type mice and ceramide species were quantified by mass spectrometry. Shown are the mean ± SD from each 6 mice. ***P < 0.001, ANOVA and post hoc Tukey test. **B** Exosomes (Exo) were isolated from the blood of untreated wild-type mice, loaded with the different ceramide species in the amount and ratio as they are present in exosomes from glucocorticosterone-treated mice and injected intravenously (i.v.) into wild-type mice. Controls (Ctrl) were exosomes from untreated mice. Neurogenesis and behavioral changes were measured as readout for the induction of MDD by i.v. injection of ceramide-loaded exosomes. Presented are the mean ± SD from each 6 mice. ***P < 0.001, ANOVA and post hoc Tukey test

We then tested whether we can induce MDD in untreated/non-stressed mice by intravenous injection of ceramide-enriched exosomes. To this end, we determined different species of ceramide in exosomes prior and after application of stress. The results indicate that in particular C22, C24, and C24:1 ceramides accumulated in exosomes after stress (Fig. 3A).

We then loaded exosomes from untreated mice with C22, C24, and C24:1 ceramide in the amount and ratio as present in stressed mice and injected them into non-stressed mice. Loading was confirmed by measuring the concentration of ceramide in the purified exosomes (not shown). We then determined biochemical and behavioral signs of MDD. These experiments showed that i.v. injection of ceramide-loaded exosomes was sufficient to induce MDD within 24 h as determined by behavioral changes and neuronal proliferation (Fig. 3B).

### Neutral sphingomyelinase 2 determines stress responses in mice

**Fig. 4:**
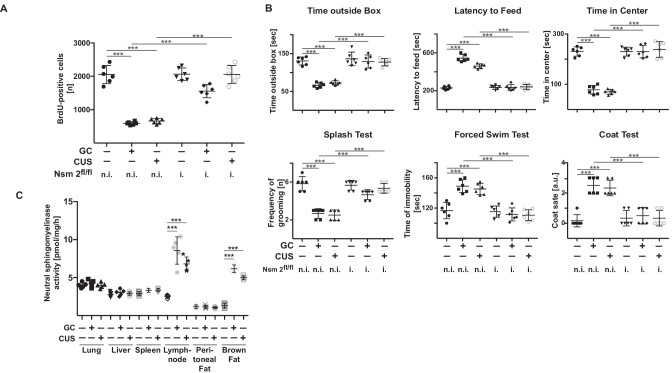
Mice with induced deletion of neutral sphingomyelinase 2 are resistant to stress induced major depressive disorder. Mice induced to be deficient for neutral sphingomyelinase 2 (i.-Nsm 2^fl/fl^) and not induced control littermates (n.i.-Nsm 2^fl/fl^) were stressed with either glucocorticosterone (GC) or chronic unpredictable environmental stress (CUS) or were left untreated. Neurogenesis **A** and behavioral changes **B** were measured to analyze major depressive disorder. Displayed are the mean ± SD from each 6 animals. ***P < 0.001, ANOVA and post hoc Tukey test. **C** Neutral sphingomyelinase activity was determined in extracts of lung, liver, spleen, lymph nodes, peritoneal fat, and brown fat. Samples were normalized for protein. Displayed are the mean ± SD from each 5 animals. ***P < 0.001, ANOVA and post hoc Tukey test

As described above, the increase of ceramide concentrations in the blood of stressed mice requires the expression of neutral sphingomyelinase 2. To confirm the role of neutral sphingomyelinase 2 in the pathogenesis of MDD, we stressed mice induced for deficiency in neutral sphingomyelinase 2 or wild-type mice with glucocorticosterone or chronic unpredictable environmental stress for each 7 days or left them untreated. The results show that various forms of stress did not induce changes in behavior and only slightly reduced neurogenesis in mice induced for deficiency of neutral sphingomyelinase 2, whereas they exerted strong effects in non-induced littermates (Fig. 4A and B).

To gain some insight into the question in which cell stress regulates neutral sphingomyelinase 2, we determined neutral sphingomyelinase 2 activity in the liver, spleen, lymph nodes, lung, yellow peritoneal fat, and brown fat tissue prior and after glucocorticosterone and CUS stress. The results indicate a marked activation of neutral sphingomyelinase 2 in immune cells (lymph nodes) and in brown fat upon glucocorticosterone and CUS treatment (Fig. 4C). Neutral sphingomyelinase 2 activity did not change in other tissues (Fig. 4C).

### Ceramide-enriched exosomes inhibit phospholipase D and reduce phosphatidic acid concentrations in the hippocampus

**Fig. 5:**
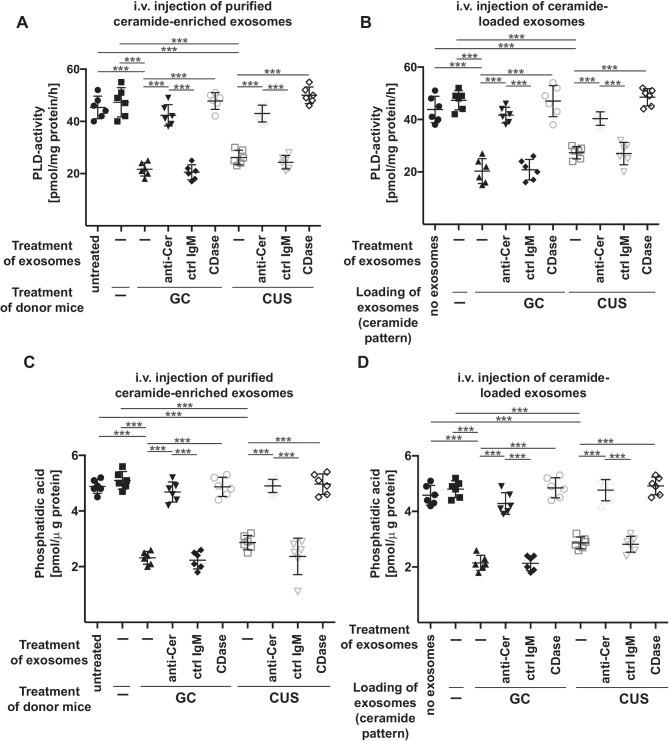
Intravenous injection of ceramide-enriched exosomes isolated from stressed mice or ceramide-loaded exosomes inhibits PLD and reduces phosphatidic acid in the hippocampus. **A** and **C** Wild-type mice were left untreated (-) or stressed with glucocorticosterone (GC) or chronic unpredictable stress (CUS) and exosomes were isolated. Purified exosomes were treated ex vivo with ceramide IgM antibodies clone S58-9 (anti-Cer), control immunoglobulin M (ctrl IgM), or recombinant ceramidase (CDase) or were left untreated (-), pelleted by ultracentifugation, resuspended in H/S, and injected intravenously (i.v.) into healthy wild-type mice. Control mice (Ctrl) were completely left untreated (-). PLD activity **A** and phosphatidic acid concentrations **C** in the hippocampus were determined 12 h after i.v. injection of exosomes. **B** and **D** Exosomes were purified from the blood plasma of untreated mice, loaded with C22, C24, and C24:1 ceramide in the amounts and ratio as these ceramide species are present in exosomes isolated from glucocorticosterone (GC)-treated mice or in exosomes isolated from mice treated with chronic unpredictable stress (CUS), washed and then i.v. injected into untreated wildtype mice. PLD activity **B** and phosphatidic acid concentrations **D** in the hippocampus were determined 12 h after i.v. injection of exosomes. Shown are the mean ± SD from each 6 animals or experiments/group. ***P < 0.001, ANOVA and post hoc Tukey test

We have previously shown that ceramide in the blood plasma inhibits endothelial phospholipase D and the formation of phosphatidic acid in the hippocampus. The lack of phosphatidic acid in the hippocampus induced MDD, which was prevented by injection of phosphatidic acid [33]. Thus, to elucidate the molecular mechanisms how ceramide-enriched exosomes mediate MDD, we tested whether injection of ceramide-enriched exosomes isolated from the blood plasma of stressed mice or of ceramide-loaded exosomes isolated from untreated mice results in a downregulation of PLD activity in the hippocampus. The results demonstrate that i.v. injection of purified ceramide-enriched (Fig. 5A) or ceramide-loaded (Fig. 5B) exosomes inhibited PLD activity in the hippocampus (Fig. 5A and B). Treatment of exosomes with anti-ceramide antibodies or ceramidase prior to injection prevented the effect of purified ceramide-enriched or ceramide-loaded exosomes on PLD-activity (Fig. 5A and B). PLD activity results in the formation of phosphatidic acid and consistent with PLD inhibition in mice injected with purified ceramide-enriched or ceramide-loaded exosomes, we observed a marked reduction of phosphatidic acid concentrations in the hippocampus of these mice (Fig. 5C and D). Intravenous injection of exosomes pretreated with anti-ceramide antibodies or ceramidase prevented the reduction of hippocampal phosphatidic acid concentrations in these mice (Fig. 5C and D).

**Fig. 6:**
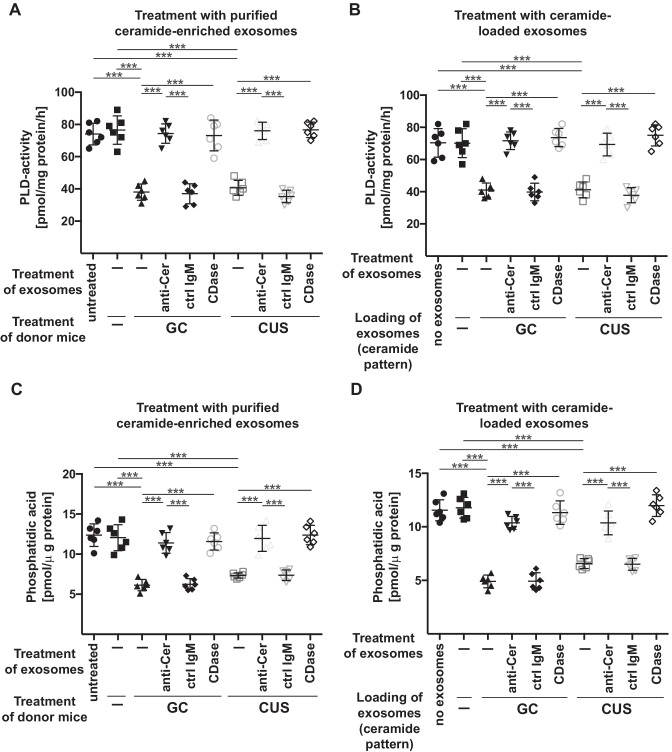
Incubation of endothelial cells with exosomes isolated from stressed mice or with ceramide-loaded exosomes inhibit PLD and reduce phosphatidic acid. **A** and **C** bEnd.3 cells were incubated with exosomes isolated from the blood plasma of wild-type mice, which were left untreated (-) or stressed with glucocorticosterone (GC) or chronic unpredictable stress (CUS). Purified exosomes were treated ex vivo with ceramide IgM antibodies clone S58-9 (anti-Cer), control immunoglobulin M (ctrl IgM), or recombinant ceramidase (CDase) or were left untreated (-), pelleted by ultracentifugation, resuspended in H/S and added to bEND3 endothelial cells. Control mice (Ctrl) were completely left untreated (-). Cells were incubated for 2 h and endothelial PLD activity **A** and endothelial phosphatidic acid concentrations **C** were determined. **B** and **D** Exosomes were purified from the blood plasma of untreated mice, loaded with C22, C24, and C24:1 ceramide in the amounts and ratio as these species are present in exosomes isolated from glucocorticosterone (GC)-treated mice or in exosomes isolated from mice treated with chronic unpredictable stress (CUS) and washed. bEnd3 endothelial cells were incubated with these exosomes for 2 h and PLD activity **B** and phosphatidic acid concentrations **D** in endothelial cells were determined. Shown are the mean ± SD from each 6 experiments/group. ***P < 0.001, ANOVA and post hoc Tukey test

In vitro studies revealed similar results: We treated cultured mouse brain endothelial bEnd3 cells with ceramide-enriched exosomes purified from blood plasma of stressed mice or ceramide-loaded exosomes isolated from blood plasma of untreated mice. This treatment resulted in a reduction of PLD activity (Fig. 6A and B) and phosphatidic acid levels (Fig. 6C and D).

**Fig. 7:**
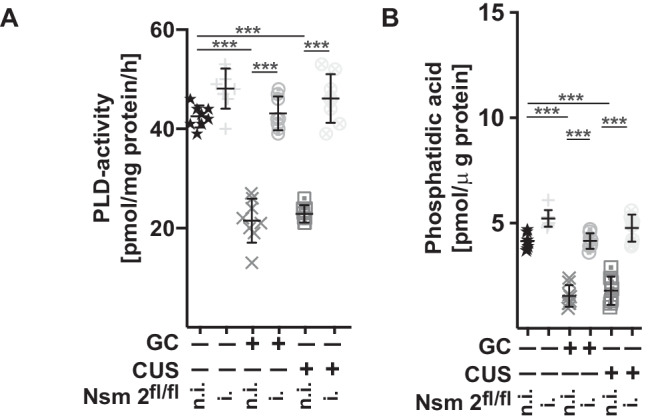
Mice with induced deletion of neutral sphingomyelinase 2 are resistant to stress-mediated inhibition of PLD and phosphatidic acid in the hippocampus. Mice induced to be deficient for neutral sphingomyelinase 2 (i.-Nsm 2^fl/fl^) and not induced control littermates (n.i.-Nsm 2^fl/fl^) were stressed with either glucocorticosterone (GC) or chronic unpredictable environmental stress (CUS) or were left untreated. Deletion of neutral sphingomyelinase 2 was induced by tamoxifen injection. PLD activity **A** and phosphatidic acid concentrations **B** in the hippocampus were determined. Displayed are the mean ± SD from each 8 animals. ***P < 0.001, ANOVA and post hoc Tukey test

Next, we tested whether induced deficiency of neural sphingomyelinase 2 protects mice from a stress-induced inhibition of PLD and reduction of phosphatidic acid in the hippocampus. Induced expression of Cre-recombinase in CreER mice resulted in a reduction of neutral sphingomyelinase activity in spleen extracts from 3.1 ± 0.28 to 0.7 ± 0.32 pmol/mg protein/h, in liver extracts from 2.8 ± 0.27 to 0.5 ± 0.19 pmol/mg protein/h and in lung extracts from 4.2 ± 0.26 to 0.8 ± 0.29 pmol/mg protein/h. The results show that genetic deficiency of neutral sphingomyelinase 2 protected mice from glucocorticocsterone- or chronic unpredictable stress-induced inhibition of PLD activity and reduction of phosphatidic acid levels in the hippocampus (Fig. 7A and B), consistent with the findings that deficiency of neutral sphingomyelinase 2 protects from signs of MDD in mice (see Fig. 4A and B).

We also determined the concentration of ceramide in hippocampus and the pre-frontal cortex in mice stressed with glucocorticosterone or CUS or injected with ceramide-loaded exosomes. The results indicate that these treatments did not significantly alter ceramide concentrations in the hippocampus or prefrontal cortex (not shown), consistent with previous studies (33).

## Discussion

The present study provides evidence for a novel concept of the pathogenesis of MDD and suggests several novel potential treatment options. We demonstrate that stress causing MDD symptoms in mouse models results in the release of ceramide-enriched exosomes into the peripheral blood of mice. Intravenous injection of exosomes from stressed mice with MDD into non-stressed mice is sufficient to induce behavioral and biochemical changes indicating and typical for MDD. Neutralizing ceramide-enriched exosomes within the blood plasma by pre-treatment with anti-ceramide antibodies or ceramidase before intravenous injection prevents MDD in non-stressed mice. Vice versa, injecting exosomes from untreated, healthy mice that are loaded with ceramide into non-stressed mice is sufficient to induce MDD, a finding indicating the importance of ceramide in blood plasma exosomes for the pathogenesis of MDD.

We show that peripheral ceramide-loaded exosomes inhibit PLD in the hippocampus and thereby reduce the basal release of phosphatidic acid in the hippocampus. We assume that ceramide-enriched exosomes are taken up by endothelial cells and thereby inhibit endothelial PLD followed by an altered crosstalk between endothelial cells and neurons. This crosstalk seems to be mediated, at least in part, by phosphatidic acid.

Ceramide has been previously shown to interact with monoaminergic and glutamate neuro-transmission [34–37]. However, we clearly show that it is peripheral, i.e., outside of the central nervous system, neutral sphingomyelinase 2 and peripheral ceramide that are increased and important in the pathogenesis of MDD. Furthermore, ceramide did not increase in the hippocampus or the prefrontal cortex after glucocorticoid stress and chronic unpredictable stress. These data suggest that ceramide does not necessarily directly act on neurons in the pathogenesis of MDD. This is consistent with our findings that ceramide inhibits PLD in endothelial cells of the hippocampus resulting in a reduction of phosphatidic acid in the hippocampus.

Phosphatidic acid has been previously shown to impact neurotransmission and to be involved in synapse functions [38]. This supports the hypothesis that the effects of peripheral ceramide are mediated by phosphatidic acid in the CNS.

Neutral sphingomyelinase 2 has been previously shown to mediate the release of exosomes [39, 40]. It is known that the enzyme is regulated by redox mechanisms [41], but the enzyme has not been linked to glucocorticosterone- or chronic unpredictable environmental stress. It might be possible that these forms of stress alter the redox metabolism in cells that respond to stress and mediate the release of ceramide-enriched exosomes, but this needs to be tested in future studies.

It is interesting to note that stress induces an activation of neutral sphingomyelinase in lymphocytes and in brown fat. The involvement of lymphocytes in MDD is paralleled by previous data showing that anxiety in mice upon application of electronic foot shocks is mediated by an increased synthesis of purines in CD4^+^ T cells [42]. Peripheral xanthine derived from CD4^+^ T cells was shown to act in amygdala and thereby to induce anxiety [42], similar to the present concepts. This is another example of a CNS symptom, i.e., anxiety that is caused by the communication of peripheral cells with the CNS. Of note, the purine-mediated mechanism seems to be independent of ceramide-mediated induction of MDD, since corticosterone-stress did not induce purine synthesis [42].

Activation of neutral sphingomyelinase 2 in brown fat tissue may link MDD to energy metabolism, but the details need further studies.

Our findings show that the corticosterone-mediated or chronic unpredictable environmental stress-induced increase of ceramide concentrations in the blood plasma requires the expression of neutral sphingomyelinase 2. In accordance, neutral sphingomyelinase 2 has been previously shown to be central for the release of exosomes [39]. It is unlikely that acid sphingomyelinase mediates the increase of ceramide levels in exosomes upon application of stress, because mice deficient in acid sphingomyelinase respond to stress with MDD [22].

The release of exosomes is also determined by the degradation of multivesicular bodies by lysosomes [43]. Thus, an impairment of lysosomal functions enhances the release of exosomes. In fact, chronic unpredictable environmental stress has been shown to impair lysosomal functions [44], but it is unknown whether this effect of chronic unpredictable stress contributes to exosome release. Glucocorticoids do not seem to induce gross changes of lysosomes [45] suggesting that the release of exosomes upon stress does not depend on a lysosomal dysfunction.

Since human individuals respond differentially to stress, it might be possible that different expression levels of neutral sphingomyelinase 2 confer resistance to stress and mediate resilience.

In addition, several inhibitors of neutral sphingomyelinase 2 are available for preclinical studies and it will be very interesting to study the effect of these drugs on the development of experimental MDD. However, these studies are beyond the focus of the present study.

Our studies indicate a central role of phosphatidic acid as effector of exosomal ceramide in the blood plasma in the pathogenesis of MDD. However, exosomes have been shown to also contain miRNA molecules that can be altered under stress [46]. Furthermore, several miRNA molecules, for instance miR-34a, miR-106, miR-134, and miR-132, are deregulated in MDD and show brain enrichment in MDD [47, 48]. It is therefore possible that, in addition to an inhibition of PLD and a reduction of phosphatidic acid, ceramide also facilitates the uptake of miR-34a, miR-106, miR-134, and miR-132 and other miRNAs into the brain [49], a mechanism that may contribute to the development of MDD.

The present study demonstrates an increase of ceramide in the blood plasma of stressed mice. Ceramide induces MDD and neutralization or consumption of ceramide prevents development of MDD upon stress. Moreover, even the injection of exosomes that are loaded with ceramide in the ratio and amounts present in the blood plasma of patients with MDD induces the development of MDD symptoms [33]. However, an increase of ceramide in the blood plasma has been also shown in other diseases, which often associate with MDD, but are certainly also independent of MDD [31–33, 50–52]. Thus, for instance, an increase of ceramide in the blood plasma has been described in patients with cardiovascular diseases, diabetes, or Morbus Parkinson [50–52]. This certainly raises the question why an increase of blood plasma ceramide can be involved in different diseases: First, the increase of ceramide might be necessary to induce for instance MDD, but not sufficient. Ceramide may only induce MDD, if additional factors are present (factors A + B + C are required to induce disease XY, while factors A + E + F are required to induce disease XX). Second, we observed a massive increase of blood plasma ceramide concentrations and the concentrations is higher than those that have been observed for diabetes of Parkinson disease or [51, 52]. Higher concentrations of ceramide might be necessary to block phospholipase D in hippocampus endothelial cells and, thus, necessary and sufficient to induce MDD. Third, the ratio of different ceramide in the blood plasma might be very important in the induction of specific effects. We mainly observed an increase of C24 and C24:1 ceramide species in MDD, while C18, C20, and C24:1 ceramide species were shown to be increased in diabetes [51]. It is presently unknown how specific ceramide species or a specific mixture of ceramide species mediates specific biological effects, but it is very likely that these differences result in different pathophysiological processes and diseases. Finally, previously, we have shown that an injection of ceramide into the blood is able to induce MDD [33]. In these studies, we injected a relatively high dose of ceramide resulting in a rapid increase of ceramide in the blood plasma and a strong inhibition of PLD in endothelial cells. Thus, if the stress mediated by ceramide is strong enough, ceramide might be able to induce MDD by its own, although additional factors are very likely required to initiate MDD under “normal” stress.

In summary, we suggest that ceramide is necessary, but not sufficient to induce MDD and it seems to require additional changes to induce MDD.

Our findings suggest that targeting neutral sphingomyelinase 2 might be beneficial to treat and/or prevent MDD. Unfortunately, at present no drugs targeting neutral sphingomyelinase 2 are available for clinical treatment, but it might be interesting to develop these drugs.

In summary, our findings provide evidence that blood plasma ceramide-enriched exosomes play a central role in the pathogenesis of MDD and suggest a novel mechanism for the pathogenesis of MDD.

## Methods

### Mice and treatments

All studies were approved by the State Agency for Nature, Environment and Consumer Protection (LANUV) NRW, Recklinghausen, Germany, # 81–02.04.2017.A084, # 81–02.04.2019.A211, # 81–02.04.2018.A413, and 81–02.2019.A003 and the local IACUC.

In the present study, we used female C57BL/6 mice wild-type mice and inducible neutral sphingomyelinase 2 (*Smpd3*) on a C57BL/6 background at an age of 8–12 weeks.

Glucocorticosterone (Sigma, Deisenhofen, Germany) was administered via the drinking water at 100 mg/L for 7 days. In the chronic unpredictable stress model, the mice were challenged for 7 days with unpredictable environmental stress, i.e., a reversal of the light/dark cycle, 3 h of 45° tilting of the cage twice each week, shaking at 125 rpm for 45 min, food deprivation for 14 h, predator sounds for 15 min, or wet cages for 1 h with two forms of stress per day in a randomized (unpredictable) order. Mice were sacrificed at day 7 after initiation of the stress. Blood was collected from the heart into heparin-coated needles and tubes immediately after the mice were sacrificed by cervical dislocation, centrifuged at 1300 × g for 5 min at 4 °C in an Eppendorf centrifuge; the plasma was removed and shock frozen in liquid nitrogen.

Embryonic stem cells allowing inducible deletion of neutral sphingomyelinase 2 (*Smpd3*) were obtained from the European Mouse Mutant Cell Repository (Helmholtz Center Munich, Germany). The gene symbol of the mice is *Smpd3*^*tm1a(EUCOMM)Hmg*^. Transgenic mice were generated, the Flp site was deleted resulting in a mouse strain in which exon 2 of *Smpd3* is flanked by two lox sites. The mice were crossed with CreEr mice (Taconic, Cologne, Germany) and deletion of exon 2 was induced by 3 intraperitoneal injections (every 2nd day) of 100 μg/25 g body weight tamoxifen. Floxed mice (littermates) that were injected with the solvent, corn oil, only were used as controls.

### Exosomes

To isolate exosomes from blood plasma, we centrifuged samples at 16,500 × g for 20 min. The supernatant was then passed through a 0.22-μm filter, and exosomes were harvested by centrifugation twice at 100,000 × g for 70 min. The final pellet was resuspended in phosphate-buffered saline (PBS). Exosomes were pelleted by centrifugation at 100,000 × g for 70 min, the supernatant was discarded, and the exosomes were resuspended and injected i.v. into healthy mice.

Exosomes in the blood plasma were quantified after ultracentrifugation using an ELISA kit against CD63 following exactly the instructions of the vendor (SBI System Biosciences, Exosome Antibodies and Elisa Kits).

Ceramide in exosomes was in vitro neutralized by incubation of the exosomes that were purified from 150 μL blood plasma as above with 0.5 μg purified IgM anti-ceramide antibody clones S58-9 or 0.5 μg control IgM or 1 μg recombinant neutral ceramidase for 45 min at 37 °C. Exosomes were again purified, the supernatants containing unbound anti-ceramide antibodies or ceramidase removed, exosomes were resuspended in PBS, and i.v. injected.

### Measurement of ceramide by kinase assay

Exosomes were purified from 50 μL plasma, resuspended in 200 μL H_2_O and extracted it in 600 μL CHCl_3_:CH_3_OH:1 N HCl (100:100:1, v/v/v). The lower phase was collected, dried, and resuspended in 20 μL of a detergent solution (7.5% [w/v] n-octyl glucopyranoside and 5 mM cardiolipin in 1 mM diethylenetriamine-pentaacetic acid [DTPA]), bath sonicated for 10 min to obtain micelles and the kinase reaction was started by addition of 70 μL of a reaction mixture containing 10 μL diacylglycerol (DAG) kinase (GE Healthcare Europe, Munich, Germany), 0.1 M imidazole/HCl (pH 6.6), 0.2 mM DTPA, 70 mM NaCl, 17 mM MgCl_2_, 1.4 mM ethylene glycol tetraacetic acid, 2 mM dithiothreitol, 1 µM adenosine triphosphate (ATP), and 5 μCi [^32^P]γATP. The kinase reaction was performed for 60 min at room temperature under shaking at 300 rpm. The reaction was terminated by the addition of 1 mL CHCl_3_:CH_3_OH:1 N HCl (100:100:1, v/v/v), 170 μL buffered saline solution (135 mM NaCl, 1.5 mM CaCl_2_, 0.5 mM MgCl_2_, 5.6 mM glucose, and 10 mM HEPES; pH 7.2), and 30 μL of a 100 mM ethylenediaminetetraacetic acid (EDTA) solution. The samples were vortexed, phases were separated, and the lower phase was collected, dried, and separated on Silica G60 thin-layer chromatography (TLC) plates with chloroform/acetone/methanol/acetic acid/H_2_O (50:20:15:10:5, v/v/v/v/v) and developed with a Fuji phosphorimager. Ceramide levels were determined by comparison with a standard curve; C16/18 and C22/24 ceramides were used as substrates.

To determine ceramide in the hippocampus and the prefrontal cortex, the brain was prepared and the hippocampus and the prefrontal cortex were carefully dissected. The tissues were homogenized in H_2_O and extracted and analyzed as above.

### Ceramide quantification by liquid chromatography tandem-mass spectrometry (LC–MS/MS)

Samples were subjected to lipid extraction with 1.5 mL methanol/chloroform (2:1, v/v) that contained d18:1/17:0 ceramide (C17 Cer; Avanti Polar Lipids, Alabaster, USA) as internal standard. Extraction was facilitated by incubation at 48 °C with gentle shaking (120 rpm) overnight. To reduce interference from plasma glycerolipids, samples were saponified with 150 µL 1 M methanolic KOH for 2 h at 37 °C with gentle shaking (120 rpm) followed by neutralization with 12 µL glacial acetic acid. After centrifugation at 2200 g for 10 min at 4 °C, organic supernatants were evaporated to dryness using a Savant SpeedVac concentrator (Thermo Fisher Scientific, Dreieich, Germany). Dried residues were reconstituted in 200 µL acetonitrile/methanol/water (47.5:47.5:5 (v:v:v), 0.1% formic acid) and subjected to LC–MS/MS ceramide quantification applying the multiple reaction monitoring (MRM) approach. Chromatographic separation was achieved on a 1260 Infinity HPLC (Agilent Technologies, Waldbronn, Germany) equipped with a Poroshell 120 EC-C8 column (3.0 × 150 mm, 2.7 µm; Agilent Technologies) guarded by a pre-column (3.0 × 5 mm, 2.7 µm) of identical material. MS/MS analyses were carried out using a 6490 triple-quadrupole mass spectrometer (Agilent Technologies) operating in the positive electrospray ionization mode (ESI +). Chromatographic conditions and settings of the ESI source and MS/MS detector have been published elsewhere [53]. The following mass transitions were recorded (qualifier product ions in parentheses): *m/z* 520.5 → 264.3 (282.3) for C16 Cer, *m/z* 534.5 → 264.3 (282.3) for C17 Cer, *m/z* 548.5 → 264.3 (282.3) for C18 Cer, *m/z* 576.6 → 264.3 (282.3) for C20 Cer, *m/z* 604.6 → 264.3 (282.3) for C22 Cer, *m/z* 630.6 → 264.3 (282.3) for C24:1 Cer, and *m/z* 632.6 → 264.3 (282.3) for C24 Cer. Peak areas of Cer subspecies, as determined with MassHunter software (Agilent Technologies), were normalized to those of the internal standard (C17 Cer) followed by external calibration in the range of 1 fmol to 50 pmol on column. Ceramide quantities were normalized to the actual protein content used for lipid extraction. Ceramide levels are expressed as individual subspecies C16, C18, C20, C22, C24, and C24:1 or as the sum of these.

### Loading of exosomes with ceramide

Exosomes isolated from 200 μL blood plasma as above were loaded with C22, C24, and C24:1 ceramide (Avanti Polar Lipids, USA) according to the mean of the amounts of the ceramides in pmol per mg protein in exosomes as determined for each ceramide species by mass spectrometry. We subtracted the amount of each ceramide species (in pmol/mg) present in exosomes from untreated mice from the corresponding values for glucocorticosterone- or CUS-treated mice, respectively, to obtain the amounts for loading the exosomes. The values were as follows: Mean ± SD of C22 ceramide from untreated mice was 12.5 ± 3 pmol/mg protein, C22 ceramide from glucocorticosterone-treated mice was 43.3 ± 9.8 pmol/mg, and C22 from CUS-treated mice was 33.1 ± 13.7 pmol/mg. The mean ± SD of C24 ceramide from untreated mice was 39.3 ± 10.8 pmol/mg protein, C24 ceramide from glucocorticosterone-treated mice was 284.3 ± 25.9 pmol/mg, and C24 from CUS-treated mice was 102.4 ± 14.3 pmol/mg. The mean ± SD of C24:1 ceramide from untreated mice was 17.6 ± 7.6 pmol/mg protein, C24:1 ceramide from glucocorticosterone-treated mice was 74.9 ± 14.3 pmol/mg, and C24:1 from CUS-treated mice was 53.4 ± 6.9 pmol/mg. We determined the protein concentration in an aliquot (25% of the total samples) of the exosomes. We then incubated the remaining exosomes (corresponding to 150 μL blood plasma) with 31 pmol/mg protein C22 ceramide + 145 pmol/mg C24 ceramide + 57 pmol/protein C24:1 ceramide to obtain exosomes that mimic exosomes released after treatment with glucocorticosterone and 21 pmol/mg protein C22 ceramide + 63 pmol/mg C24 ceramide + 36 pmol/protein C24:1 ceramide to obtain exosomes that mimic exosomes released after CUS. Ceramides were suspended in 0.9% NaCl and sonicated prior to use to get a homogenous suspension. Incubations of purified exosomes with ceramides were performed in 100 μL PBS for 60 min at 37 °C. Exosomes were centrifuged again at 100,000 × g for 70 min; the supernatant was discarded, resuspended in PBS, and injected i.v. into healthy mice.

### Neutral sphingomyelinase activity

Mice were stressed with glucocorticosterone or chronic unpredictable stress or left untreated as above, sacrificed; the lung, liver, spleen, lymph nodes, yellow peritoneal fat, and brown interscapular fat were removed. Tissues were shock frozen, homogenized in 100 mM HEPES (pH 7.4), 5 mM MgCl_2_, 1% NP40, and each 10 μg/mL aprotinin/leupeptin using a tip sonicator, and aliquots were diluted tenfold in 100 mM HEPES (pH 7.4), 5 mM MgCl_2_, 0.2% NP40, and each 10 μg/mL aprotinin/leupeptin. The assay was started by addition of 0.05 μCi [^14^C]sphingomyelin (52 mCi/mmol; Perkin Elmer, #NEC 663010UC) per sample in 30 μL 100 mM HEPES (pH 7.4), 5 mM MgCl_2_, 0.2% NP40, and each 10 μg/mL aprotinin/leupeptin. The substrate [^14^C]sphingomyelin was dried for 10 min in a SpeedVac, resuspended in 100 mM HEPES (pH 7.4), 5 mM MgCl_2_, 0.2% NP40, and each 10 μg/mL aprotinin/leupeptin and sonicated for 10 min in a bath sonicator before it was added to the samples. The samples were incubated for 60 min at 37 °C with shaking at 300 rpm. Samples were then organically extracted in 4 volumes of CHCl_3_:CH_3_OH (2:1, v/v), vortexed, the samples were centrifuged, and an aliquot of the upper aqueous phase was scintillation-counted to determine the release of [^14^C]phosphorylcholine from [^14^C]sphingomyelin.

### Immunohistochemical bromodeoxyuridine staining

Bromodeoxyuridine (BrdU, 2 mg/25 g body weight) was intraperitoneally injected 3-times, once every 2 h, at a dose of 75 mg/kg, starting 16 h before the mice were sacrificed. Mice were euthanized and perfused via the left heart for 2 min with 0.9% NaCl followed by a perfusion with 4% paraformaldehyde (PFA) buffered in PBS (pH 7.3) for 15 min. The brains were removed, fixed for an additional 36 h in 4% buffered PFA in PBS, embedded in paraffin. Paraffin-embedded sections were dewaxed, treated for 20 min with pepsin at 37 °C, washed, incubated for 2 h with 50% formamide in 300 mM NaCl and 30 mM sodium citrate (pH 7.0) at 65 °C, and washed twice in saline sodium citrate buffer. DNA was denatured for 30 min at 37 °C with 2 M HCl, washed, neutralized for 10 min with 0.1 M borate buffer (pH 8.5), washed, and blocked with 0.05% Tween 20 and 5% FCS in PBS (pH 7.4). The samples were then stained for 45 min at 22 °C with 5 μg/mL BrdU-specific antibodies (Roche, Mannheim, Germany, # 111,703,760,001), washed, and stained with Cy3-coupled F(ab)_2_ anti-mouse IgG antibody fragments (Jackson ImmunoResearch, West Grove, PA).

All sections were analyzed with a LEICA TCS SL fluorescence confocal microscope. Every tenth section of serial sections of the hippocampus was counted by an investigator blinded to the nature of the samples and the number of BrdU positive cells was calculated.

### Endothelial cells

bEND3 cells (ATCC; LGC Standards, Wesel, Germany) were cultured in DMEM, supplemented with 10% fetal calf serum, 10 mM penicillin/streptomycin, 1 mM sodium pyruvate, an 2 mM L-glutamine. The cells (50,000 cells per sample) were incubated for 120 min with exosomes isolated from 100 μL blood plasma as above or left untreated. To determine PLD activity in endothelial cells, the medium was removed, the cells washed, homogenized, and the enzyme activity was measured as described below.

### PLD activity

Phospholipase D activity was measured employing a commercial colorimetric activity assay (Abcam, #ab183306). To determine PLD in the hippocampus, the hippocampus was removed, homogenized, and incubated in assay buffer on ice for 15 min, insoluble material was removed by 5-min centrifugation at 14,000 rpm at 4 °C, and an aliquot of the lysate was mixed with the PLD substrate, the PLD probe, and the converter enzyme to generate the colored substrate according to the instructions of the vendor. Each sample was measured against a background sample that contained the same amount of lysate and reagents with the omission of PLD substrate. Samples were analyzed at OD 570 nm in a microplate reader (Fluostar Omega, BMG Labtech) in kinetic mode every 2 min. The enzyme activity was calculated and normalized for protein.

### Phosphatidic acid analysis

Phosphatidic acid was measured in hippocampus extracts and in endothelial cells extracts using a commercial assay (PromoKine, #PK-CA577-K748) exactly following the protocol of the vendor. Hippocampus was removed, homogenized by tip sonication in 200 μL H_2_O and extracted in 750 μL CHCl_3_:CH_3_OH:12 N HCl (2:4:0.1, v:v:v) and each 250 μL CHCl_3_ and 1 M NaCl. Samples were vortexed and centrifuged for 5 min at 14,000 rpm to separate phases, the organic phase was dried in a SpeedVac, samples were dissolved in 5% Triton X-100, and the reaction was started by addition of a phosphatidic acid converter. The converter hydrolyzes phosphatidic acid to an intermediate that is converted to a fluorescence substrate in the presence of the provided enzyme mix. Samples were analyzed in a fluorescence reader at excitation/emission of 535/587 nm. The amount of phosphatidic acid was calculated by comparison to a standard curve.

### Protein measurements

Protein was measured employing the BioRad Protein Assay Dye (#500,006) from aliquots of the hippocampus homogenates or lysates or exosome preparations to normalize the samples.

### Behavioral studies

Behavioral testing was performed between 3:00 p.m. and 6:00 p.m. under diffuse indirect room light. All tests were performed on separate days. If appropriate, animals were tracked with a video camera (Noldus Systems, Worpswede, Germany). For the *novelty-suppressed feeding* test (latency to feed), mice were fasted for 24 h, placed in a new environment containing one piece of food on a small piece of white paper in the middle of the arena, and the time until mice began eating was recorded. For the *light/dark box test*, mice were placed in a dark and safe compartment that was connected via a 5-cm × 5-cm rounded-corner aperture to an illuminated, open, and thus aversive area. The time that the mouse spent in each of the separate compartments was measured. In the *open-field arena* test, the mice were released near the wall of a 50-cm × 50-cm white plastic cage with sidewalls 30 cm high. Animals were observed for 30 min, and the time during which the animal was more than 10 cm away from the wall was recorded. In the *coat state* test, the appearance of the coat (groomed vs unkempt coat) was scored on the head, neck, back, and ventrum with either 0 for normal status or 1 for unkempt status. For the *forced swim test*, mice were placed in a cylinder filled with water (21–23 °C) for 15 min. After 24 h, the mice were again placed in a water-filled cylinder for 6 min, and the time of mobility or immobility during the last 4 min of the second trial was recorded. Mice were judged immobile when they moved only to keep their heads above water.

### Quantification and statistical analysis

Data are expressed as arithmetic means ± SD. For the comparison of continuous variables from independent groups with one variable (treatment), we used one-way ANOVA followed by post hoc Tukey test for all pairwise comparisons, applying the Bonferroni correction for multiple testing. The *P*-values for the pairwise comparisons were calculated after Bonferroni correction. We tested all values for normal distribution and similar variances. For the analysis of groups with 2 variables (treatment and genotype), we used one-way ANOVA and post hoc Tukey test for multiple comparison. Statistical significance was set at a *P*-value of 0.05 or lower (two-tailed). The sample size planning was based on the results of two-sided Wilcoxon-Mann–Whitney tests (free software: G*Power, Version 3.1.7, University of Duesseldorf, Germany).

To avoid bias during analysis, investigators were blinded to the identity results of histological samples and animals. Animals were randomly assigned to cages by a technician who was not involved in the experiments prior to the experiments. Cages were randomly assigned to the various experimental groups.

## Data Availability

All data are included in the manuscript.
